# Impact of Critical Success Factors on Project Success Through the Mediation of Knowledge Creation

**DOI:** 10.3389/fpsyg.2022.892488

**Published:** 2022-06-07

**Authors:** Saira Naseer, Kashif Abbass, Muhammad Asif, Hammad Bin Azam Hashmi, Sidra Naseer, Monica Violeta Achim

**Affiliations:** ^1^School of Economics and Management, Nanjing University of Science and Technology, Nanjing, China; ^2^Riphah School of Business and Management, Riphah International University, Lahore, Pakistan; ^3^Department of Economics & Business Administration, University of Education Lahore, Multan, Pakistan; ^4^Department of Environment Science, University of Wah, Wah Cantt, Pakistan; ^5^Department of Finance, Babes-Bolyai University, Cluj-Napoca, Romania

**Keywords:** health project success, critical success factors, knowledge creation, project, success

## Abstract

Several factors affect health project success. This research aims to examine the impact of critical success factors on health project success and show how the essential factors of success interact with knowledge creation to impact health project success. The self-administered questionnaire was distributed to collect data from 246 managers, supervisors and zonal supervisors of DHQ hospital Attock and PIMS hospital Islamabad. The analysis was done using Smart PLS to understand the effect of exogenous variables over endogenous variables and the impact of mediating variables between two constructs. The results show that all critical success factors (MGTRF, DRF, CRF, PMRF, CLRF) are significantly affecting project success, in addition, tacit knowledge creation mediate the association between critical success factors and project success. In contrast, explicit knowledge creation does not mediate the relationship between critical success factors and project success. This study intends to expand the theoretical understanding of process improvement by providing practical insights into the impact of strategies used by project managers to develop new knowledge by capturing explicit and implicit information. This study also reinforces past findings and increases awareness about using knowledge creation to gain a competitive advantage in the health sector.

## Introduction

According to a Pakistan economic review (2017–18), the government spends 0.35% of its total GDP on health care. There are currently 5,382 clinics, 1,207 hospitals, 5,404 basic health care units, and 696 child care and maternity units in Pakistan. However, Pakistan faces major health concerns due to poor and unfavorable health circumstances. According to Khan and Van Den Heuvel (2006), numerous governments in Pakistan have worked on health programmes, but the benchmark has not yet been set due to various internal factors. Pakistan also announced the “National Health Policy” last year, aiming to improve society's better health conditions. Policymakers and top management may not take health project success seriously, limiting health development in Pakistan. To resolve the above issues, the government needs to consciously take part in health issues and look at every step to complete the health services. Sheikh and Jensen (2019) results indicate that due to the poor health conditions in Pakistan, it has to suffer from various diseases in routine life. The population appears as a genetic problem on the international level; the global world focuses on genetic disorders due to health issues, how can reduce genetic diseases, and what measures are required to cope with them. According to Habib et al. (2017), polio teams are attacked by terrorists due to security concerns; thus, hospitals must take sufficient precautions for such treatments. Good planning is only possible when top management is fully aware of the issue, and knowledge gained from experience.

Knowledge has played an important role in the success of projects and is the key to innovation and competitiveness for an organization (Canonico et al., 2019; Yang et al., 2021). Studies have shown that employees working on a project acquire new knowledge (both explicit and tacit) from their encounters (Todorović et al., 2015). Excellent performance and greater project success could be achieved after creating and utilizing knowledge in the product, business process, and services. Knowledge, both explicit and tacit about the previous project leads to project success. Tactic knowledge is created through discussions with stakeholders, office colleagues, project partners, consultants, and experts. Values, beliefs, assumptions, and mental models comprise tacit knowledge. While explicit knowledge is codified, implicit knowledge is not.

A database, web pages, emails, and documents store explicit knowledge (Boon Sin et al., 2015). Whenever a problem arises in the project, it is necessary to schedule sessions with a professional. Professionals and experts share ideas with employees. Professionals acquire insights from documented information and use that knowledge for problem resolution (Canonico et al., 2019). Similarly, Japanese car manufacturing creates competitive advantage and innovation dynamically by using Nonaka et al. (2000) SECI model (Allal-Chérif and Makhlouf, 2016). We can also use this theoretical framework in health projects to analyze knowledge creation practices because health project has an important and huge impact on the nation (Sheikh and Jensen, 2019). This study is based on Nonaka theory. This study also expands the model of Todorović et al. (2015) by introducing knowledge creation as a mediator and project success (customer happiness) as a dependent variable. The project's effectiveness has been extensively discussed in the literature, but more research is needed to uncover the most influential CSFs that influence health project success (Kiani Mavi and Standing, 2018b; Maqbool and Sudong, 2018). The study investigates “the impact of CSFs on project success *via* knowledge creation mediation” (Todorović et al., 2015).

This study intends to achieve the following objective;

- To determine the effect of critical success factors on project success.
- To determine the effect of knowledge creation on project success.
- To determine the mediating role of tacit knowledge creation between the critical success factors and project success.
- To determine the mediating role of explicit knowledge creation between the critical success factors and project success.

To achieve the above objectives, this study focuses on the following issues.

- Is the critical success factors having an impact on project success?
- Is knowledge creation significantly related to project success?
- Does tacit knowledge creation mediate the effect of critical success factors on project success?
- Does explicit knowledge creation mediate the effect of critical success factors on project success?

### Significance of the Study

In this research we will find all those critical success factors that are leading project toward success. CSFs are those few area of activity that is actually causing the success of the project. These CSFs have been used in many industries before, like manufacturing industry, construction industry, information system and financial service, etc. the intensity and magnitude of different factors have been identified by different researchers, but in this study we will examine that how many and in which degree these factors are influencing a project's success. According to their importance we will also take an order of these factors. We will also examine that how the tacit and explicit dimension of knowledge creation positively influence between CSFs (MGT, Procurement, Manager, Contractor, and Stakeholder related factors) of project success. In the concluding part of the study a clearer picture has been developed that highlights the factors and their relationship with project success in accordance with the local industry view points. Through this research manager come to know about CSFs factors which affect the project success. And when these factors efficiently manage then many projects could reach the desired success. The study is significant because the top management will get awareness that their strong coordination with their subordinate are the main reason for project success. It makes the authorities realize that how they can use different strategies to handle the critical success factors in the project so that the work environment will be more friendly and its lead project toward success. It helps the policy maker to understand the impact of critical success factors on project success, so that the different policies can be made to handle these critical success factors which may affect project success badly.

This study is organized into six sections. In the first section, we shall discuss the study's context. The second section will present a high-level summary of the research, theories, and models related to project success, critical success factors, and knowledge creation. The third part will give a conceptual framework and operational definition of the variables. The fourth section discusses research methodologies, such as data gathering and the construction of metrics. The fifth portion contains the analysis, discussion of the results, study limitations, managerial implications, recommendations for future work, and study conclusion. In the study's conclusion, a clearer picture of the components and their link with project performance has been produced.

## Literature Review

### Customer Satisfaction

Customer satisfaction has been mentioned in the project management literature (Kerzner, 2002), but it has rarely been included in the formal assessment of success. The client is the one that spends the majority of their time on developed facilities, are actually working and live with the final products. It is critical to ensure that the finished project satisfies the client's/customer's expectation. As per Liu and Walker (1998), satisfaction should be regarded a success criterion. Customer functional performance, expectations, and technical specifications, as demanded by clients, are critical in project business. This rationale is that “the client is the king/boss of every business/organization/project.” As a result, client happiness is the primary goal of any firm.

A company must first meet its customers' expectations to ensure customer satisfaction. If the project satisfied the end-user (Torbica and Stroh, 2001), it could be considered effectively completed in the long run. De Wit (1986) defines project success as determining whether the aim and goal are met. Customer happiness is more vital to success than reaching any specific project objectives. Project deliverables are also critical to achieving the customer's satisfaction level. If the customer is pleased with the result/product, the project has been deemed successful.

### Critical Success Factor

Several research conducted over the last few decades has demonstrated the significance of critical success factors. Denial was the first to introduce the concept of critical success criteria in 1961. In 1982, Rock art used the term Critical Success Factors for the first time. These are “factors that contribute to an organization's success and are important for the achievement of an organization's mission,” according to Haleem et al. (2012). Rubin and Seelig (1967) pioneered the critical success factor in project management, assessing the influence of the project manager's experience on project success. A critical success factor is a business strategy. Essential success factors are related to a specific attribute or condition of the industry.

Because each country has its own set of rules and regulations, legal constraints, and operational environment, Critical Success Factors differ when we travel from one country to another and from one project to the next. Several dimensions of critical success factor have been specified in detail by several authors (Ashley et al., 1987; Pinto and Slevin, 1987; Belassi and Tukel, 1996; Eriksson, 2008; Yang et al., 2009; Ibbs et al., 2010; Ahmadabadi and Heravi, 2019). According to Saqib et al. (2008), seven critical success factor influence project success. These dimensions include design-related factors, project management-related factors, contractor related factors, economic and business environment-related factors, client related factors, procurement related factors, and program manager related factors.

In his study, the most important factors were project managers' skills, contractors' experience, contractors' cash flow, site management, prompt decisions by clients, prior management experience, solving and decision-making efficacy, supervision, and clients' decision-making capacity. The findings show a positive association between design-related factors, project management-related factors, contracting related factors, business and work environment-related factors, client-related factors, procurement related factors, project manager related factors, and project success. Many project managers may find this study valuable in analyzing the success of their current project. A project manager can evaluate the present value of their program and compare actual and projected values for considered successful elements in a knowledge management activity. It is a useful hint to encourage us to investigate these aspects in the health sector.

### Dimensions of Critical Success Factors

According to Saqib et al. (2008), the Critical Success Factor has seven dimensions.

- Design related factors
- Project Management related factors
- Contractor related factors
- Business and Work Environment related factors
- Client related factors
- Procurement related factors
- Project Managers related factors.

#### Management Related Factors

One technique to ensure project success is to meet the criteria of Hubbard's project management actions (Hubbard, 1990; Misztal-Okońska et al., 2020). Shen and Liu (2003) identified management-related critical issues. The findings highlight two important aspects of project success: “coordination among the management team and collaborative efforts by the client, contractor, and consultant.” Kiani Mavi and Standing (2018a) defined critical success factors (CSFs) in project management and classified them into five criteria groups: external environment, organization, project management, project, and sustainability. Data were collected from 26 Australian project managers in the construction industry for this study to establish the dependency and weight of the CSFs.

According to Mavi and Standing's findings, the biggest weights are allocated to top management and sponsor support, end-user imposed constraints and stakeholder expectation. Previous studies have not adequately addressed these issues. To complete the project, the project manager would organize and enforce it utilizing management tools (Jaselskis and Ashley, 1991). These elements (control mechanism, decision-making efficacy, feedback capabilities, adequate communication, risk identification and allocation, plan and schedule of project followed, and previous project management experience of related projects) are extremely important in health projects; if these factors are handled effectively, then it will ensure project accomplishment.

In a health project, project management-related factors such as “feedback capability, communication system, planning effort, organizational structure, control mechanism, control of subcontractors, safety and quality assurance programme, and finally the overall managerial actions” will impact. According to Pinto and Slevin (1987), communication and troubleshooting, monitoring and feedback, senior management support, and a timeline must all be present at all stages of the implementation process. Top management support has been identified as a critical success factor in several studies (Belassi and Tukel, 1996; Young and Jordan, 2008; Dikic et al., 2020). Another study found that top management related factors had a positive relationship with project success (Shenhar et al., 1997; Jugdev et al., 2001; Saqib et al., 2008).

*H1a: Top management related factors is positively and significantly related to project success*.

#### Client Related Factors

The project leader, advisors, consumer, vendor, operator, contractor, and manufacturers are significant project participants (Chua et al., 1999). The “client” could be either public or private. Client-related aspects include “client type and experience, project organization expertise, client characteristics, project funding, owner construction sophistication, client confidence, well-defined scope, client project management, and owner risk aversion” (Chan and Kumaraswamy, 1997; Songer and Molenaar, 1997; Dissanayaka and Kumaraswamy, 1999). The project was completed under the client's specifications. As a result, there is a need to effectively engage with the client, keep them updated frequently, and make changes to the project to be completed successfully.

Client experience, the client's capability to brief the project, the client's ability to make appropriate decisions, and the client's ability to clearly define roles are all important elements to consider in the project's completion.

It is a useful hint to encourage us to investigate these aspects in the health sector. Another study found that client-related factors were positively connected to project success (Walker, 1995; Saqib et al., 2008).

*H1b: Client related factors are positively and significantly associated with project success*.

#### Design Related Factors

According to Salmeron (2009), a project design phase is one way to satisfy the owner's requirements economically and optimally. The project's design phase can be depicted structurally. According to Chalabi and Camp (1984), adequate planning at the beginning of a project can reduce the likelihood of cost overruns and delays. As a result, the project will be completed within the time-frame indicated, and the project will be delivered to the customer as promised. Designers play an important role in a project because their work continues from completion to inception.

According to Chan and Kumaraswamy (1997), design team-related factors include project design complexity, design team experience, and mistakes and delays in providing project design documents. According to Chan and Kumaraswamy (1997), the primary cause of project delays was the consultant's lack of design experience. Inexperienced design consultants do not adequately design a project, resulting in time and cost overruns and project delays. Another study discovered that design related factors had a positive link with project success (Chalabi and Camp, 1984; Saqib et al., 2008).

*H1c: Design related factors are positively and significantly associated with project success*.

#### Contractor Related Factors

Contractors and subcontractors begin their primary responsibilities once a project is completed. A lack of relevant contractor planning and monitoring experience and a lack of design experience on the part of consultants would result in a project time overrun. The variables include: “management of the project site, subcontracting involvement and supervision, experiences of a contractor, cash flows of contractor, speed of information flow, and cost control system effectiveness” (Chan and Kumaraswamy, 1997; Dissanayaka and Kumaraswamy, 1999). Lu et al. (2008) described contractor related critical success factors; it's mainly include, insufficient contractor expertise, labor productivity, owner intervention, financing and payment, inappropriate planning, slow decision making, and subcontractors. According to Odeh and Battaineh (2002), consultants and contractors agreed that the top 10 most critical success factors are bad contractor experience, labor productivity, owner intervention, financing and payment, inappropriate planning, slow decision making, and subcontractors. When an organization can deal with these factors efficiently, it can complete a project on time, at a low cost, and with high quality for its clients. As a result, the project undertaken by the project organization is successful. Another study found that contractor-related factors (particularly cash flow and contractor experience) positively correlated with project success (Saqib et al., 2008).

*H1d: Contractor's related factors are positively and significantly related to project success*.

#### Project Manager Related Factors

The project manager is another significant stakeholder in projects. Project manager skills impact scheduling, project planning, and communication (Belassi and Tukel, 1996). The engagement and devotion of project managers are crucial for project completion and will replicate this if the project manager is overseeing many projects simultaneously. The project manager's skill and qualities, competency, devotion to the project, experience, and project authority are all project manager-related factors (Chua et al., 1999). The project manager is in charge of ensuring that the project is managed effectively and efficiently.

As a result, the project manager must be proficient in project management. Project management competency/skill, project-related experience, leadership ability, technical capabilities (with contractor and subcontractor), and reporting abilities are critical to project success. Another study revealed that project manager-related factors (especially project manager expertise) has a positive association with project success (Saqib et al., 2008).

*H1e: Project manager related factors are positively and significantly associated with project success*.

Previous research concentrated solely on the proposed idea of success analysis. For example, determining CSFs and success criteria, followed by linking CSFs and success criteria (Bhatti, 2005; Hyvari, 2006; Saqib et al., 2008). This study will look at the impact of critical success factors on project success *via* the mediation of knowledge creation. According to the extant literature, none considered the relationship between critical success criteria and project success in health sectors. Different studies were carried out in past years, and they mainly focus on the construction sectors. It has been noticed that the researcher has neglected the health sector. That is the purpose that encourages the researcher to carry out this research. The present study adds value to the extended body of literature by covering health sectors.

### Knowledge Creation

An organization's competitive advantage is derived from its knowledge resource, which is valuable, rare, and non-replaceable. In the hope of improving performance through better management of what they know, organizations have been proactively engaged in knowledge management. Knowledge management is generally defined as the ability to leverage knowledge to achieve organizational goals, although theories tend to focus either on people or on technology (Miković et al., 2020). According to Hu et al. (2019), knowledge management in a project environment is an understudied topic in project management. Knowledge is the most precious asset in the context of project management. According to Awad and Ghaziri (2004), knowledge is ”everything that can learn *via* the process of experience or suitable studies.” It is crucial to emphasize that data, information, and knowledge have always been significant in the industrial, information, or agrarian ages. Many issues confront the nation and organization; to gain a competitive advantage, the government and organization must deal with knowledge assets (Ali, 2008).

As a result, to be more competitive, inventive, and productive, organizations must manage their knowledge resources effectively and strategically. However, the dilemma of attaining the organization's goal through utilizing and producing new information arises. The knowledge managed by organization's includes both explicit and implicit information. The organization's leadership may supply all required information related to identifying, sharing, and developing knowledge. An organization requires a mechanism for knowledge generation, information sharing, and organizational learning (Rowley et al., 2000; Reich et al., 2012). Knowledge play important in the project. Knowledge of previous projects propels us to project success.

It might be either explicit or implicit. Tactic knowledge is created through discussions with stakeholders, office colleagues, project partners, consultants, and experts. It is extremely difficult to express tacit knowledge. Tactic knowledge is acquired *via* study and experience. Interaction with other individuals fosters the development of tacit knowledge. Tacit knowledge is unintentional and generally restricted to a particular region. Because it is not found in many books or manuals. Tactic knowledge comprises values, attitudes, assumptions, and mental models since it is cognitive and technical. When people use a variety of technical abilities, their tacit understanding of the technological base is articulated. Perception and implicit mental models are exhibited when tacit cognitive knowledge is employed (Sternberg, 1997).

Tacit knowledge is easier to remember than explicit knowledge (Wah, 1999). Face-to-face interaction, such as informal talk, internships, and storytelling, transforms two-thirds of work-related information. Whenever an issue arises in the project, it is necessary to arrange a meeting with an expert because project-related professionals and experts share their perspectives with employees. When tacit information is expressed, relevant decisions may resolve the problem. The project will be completed on schedule and under budget, resulting in project success. Social contact is nonetheless a requirement for tacit awareness. Social contact and replication of life and work skills can provide a platform for the production, sharing, and transmission of information (Ibrahim et al., 2021). Observations also show that social interaction can occur, especially in aggregated cultures, where people of various ethnic backgrounds and backgrounds share their feelings, perceptions, and ideas. Organizational cultures provide a system of learning, adapting and creating a strong environment for people to share valuable insights, which create added value to the organization. Organizational culture, for example, aims to control its members' actions through information sharing. In addition, organizations can create an atmosphere in which employees can make use of their cognitive skills to develop knowledge and share creative ideas. Tacit knowledge has a favorable impact on project success. So, tacit knowledge plays a mediating role between project success and success factors (Arumugam et al., 2013; Dalkir, 2013).

*H2a: Tacit Knowledge creation mediates the relationship between management-related factors and project success*.
*H2b: Tacit Knowledge creation mediates the relationship between contractor related factors and project success*.
*H2c: Tacit Knowledge creation mediates the relationship between design-related factors and project success*.
*H2d: Tacit Knowledge creation mediates the relationship between project manager related factors and project success*.
*H2e: Tacit Knowledge creation mediates the relationship between client-related factors and project success*.

Implicit information is not codified, but explicit knowledge is. May find Detailed expertise in databases, the internet, email, and papers. Everyone can easily get access to explicit knowledge and can easily share (Hansen et al., 1999). To overcome many similar problems, we use codified and recorded explicit knowledge. We urge project team members to preserve a written record of working ability, a regulated technique for keeping a record of working knowledge, and a working knowledge record in the information system.

When an employee encountered a problem on the project, project-related internal documents and data files were freely accessible/available. The employee derived ideas and used that information to solve the problem (Smith, 2001). As a result, explicit information contributes to project success. As a result, explicit knowledge plays a mediating role between project success and success factors (Arumugam et al., 2013; Dalkir, 2013; Todorović et al., 2015).

*H3a: Explicit Knowledge creation mediates the relationship between management-related factors and project success*.
*H3b: Explicit Knowledge creation mediates the relationship between contractor related factors and project success*.
*H3c: Explicit Knowledge creation mediates the relationship between design-related factors and project success*.
*H3d: Explicit Knowledge creation mediates the relationship between project manager related factors and project success*.
*H3e: Explicit Knowledge creation mediates the relationship between client-related factors and project success*.

### Conceptual Framework of the Study

**Figure 1:**
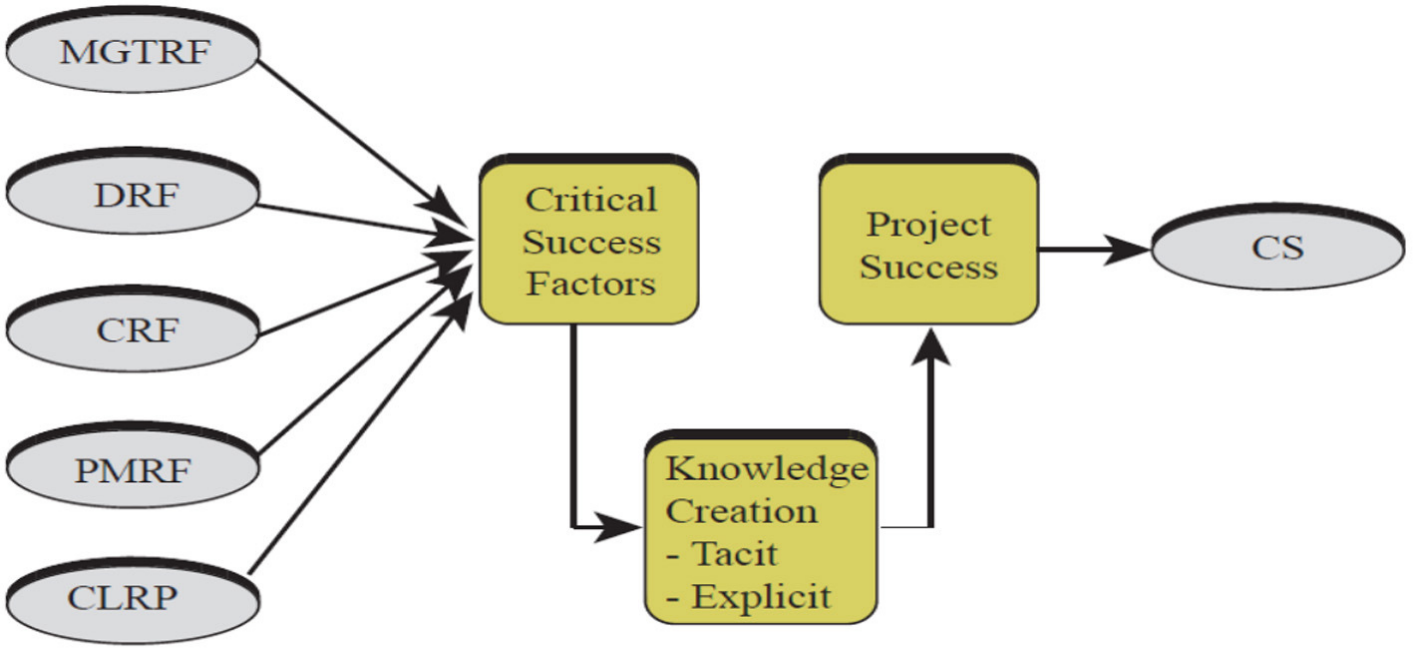
Conceptual framework. Source: Author's constructed.

Institutions that use their tacit knowledge to handle various problems and achieve their goals have a major competitive advantage (Smith, 2001). As previously stated, after examining specific projects, some writers believe that gathering of information on project results might be a great strategy to develop a knowledge base that can use to manage future projects (Hanisch et al., 2009; Yun et al., 2011). None of the above review studies has considered the relationship between knowledge creation and project success based on the existing literature. The purpose of this study is to give a framework for analyzing project success that will allow others to gather information on project results achieved in various segments and investigate the impact of the proposed idea of knowledge creation in a project environment. The research model of this current study is given in Figure 1.

## Research Methodology

The current study employed a quantitative methodology and a deductive approach. It is explanatory because it explains the variable's causal link. This study uses the deductive method, also known as testing theory, which formed the hypothesis based on theory. A cross-sectional study is employed because it is less expensive, saves time, and is the most commonly used survey approach. The current study's population was the health project of DHQ Hospital Attock and PIMS Hospital Islamabad. Due to time constraints and limited resources, the study only choose two hospitals as a population. The current study's respondents were “Manager, Supervisor, Zonal Supervisor, and LHS.”

The Manager, Supervisor, Zonal Supervisor, and LHS were chosen because they are in charge of the key functions of the health project and review various stages of health projects such as Polio, EPI, Mother-Child health care, Measles, T.B. dot programme, and so on. Furthermore, depending on their project experience, these individuals provide reliable information. The current study employs non-probability sampling, which means that every unit of the population is unknown and does not have an equal chance of being selected for the sample. There are alternative non-probability sampling procedures, but convenience sampling was used in the current investigation. It is made up of people who are easy to reach. The benefit of using this technique is that it saves time, money, and it is useful in the pilot study. For that reason this type of sampling preferred for that study. The current study's sample was selected using Morgan and Krejcie (1970) criterion, and the sample size for <800 populations is around 246.

See Table 1, the current study's data is gathered *via* a questionnaire because there is a lot of information to be collected in a short period. The questionnaire is based on a literature review and prior studies on this topic. The question was broken down into four sections. A pilot study was conducted to evaluate the instruments in the current study. According to Galloway (1997), the pilot research should be between 5 and 10% of the final sample size, and 10% of 246 equals 25.

**Table 1:** Distribution and collection of questionnaire.

**Hospital**	**Total questionnaire distributed**	**Received**
DHQ Attock	166	133
PIMS Islamabad	134	113
	300	response rate = 82%

The questionnaire was given to 30 people in the current study. Cronbach's alpha was used to determine the internal consistency of the scale. A figure in the range of 0.7 to 0.95, according to Nunnally and Bernstein (1994), is suitable. Cronbach's alpha was estimated using PLS (Partial Least Square) software for the current study. The Cronbach's alpha value of the instruments and individual constructions is shown in the table below (see Table 2), proving that the instrument is reliable. The component would be assessed using a five-point Likert scale ranging from 1 (very un important) to 5 (very important).

**Table 2:** Pilot testing results.

**Constructs**	**No. of items**	**Cronbach's alpha**
Client related factors	6	0.810
Contractor related factors	5	0.824
Design related factors	5	0.665
Knowledge creation	12	0.857
MGT related factors	7	0.900
Project manager related factors	5	0.818
Project success	6	0.838

*In this section of the study, construct of the questionnaire is briefly discussed. Following are the table, which provides detailed information about the constructs of the study (see Tables 3, 4)*.

**Table 3:** Construct and items.

**Sr**	**Variables**	**No of items**	**References**
1	Management-related factors	7	Saqib et al., 2008; Ismail et al., 2012
2	Design related factors	5	Saqib et al., 2008
3	Contractor related factors	5	Saqib et al., 2008; Doloi et al., 2011
4	Project Manager related factors	6	Saqib et al., 2008; Seiler et al., 2012; Verburg et al., 2013
5	Client related factors	7	Saqib et al., 2008
6	Knowledge creation	14	Songer and Molenaar, 1997
7	Project success	6	Shenhar et al., 1997

**Table 4:** Sources of reflective constructs.

**Reflective constructs**	**Construct items**	**References**
MGTRF	Communication system among all members.	Saqib et al., 2008; Ismail et al., 2012
	The control mechanism of project activity.	
	The feedback system for all Stakeholders.	
	Planning ability of project team.	
	Decision-making ability of Project team.	
	Prior project management experience of the project team.	
	The ability of the project team of risk identification and allocation.	
DRF	Design team experience [design experience of consultant (Architect/Engineers)].	Saqib et al., 2008
	Project design complexity.	
	Mistakes/delays in producing design documents.	
	Design team's contribution to project.	
	Adequacy of plans and specifications.	
CRF	Contractor experience of related projects.	Saqib et al., 2008; Doloi et al., 2011
	The ability of a contractor to supervise project activity.	
	The ability of the project manager to supervise the contractor.	
	The ability of the project team to manage contractor case flow.	
	Speed of information between project organization and contractor.	
PMRF	Project manager competence/skill.	Saqib et al., 2008; Seiler et al., 2012;
	Project manager experience related to project.	Verburg et al., 2013
	Leadership skills of project manager.	
	Technical capability of project manager.	
	Organizing and coordinating skills of project manager with contractor and subcontractor.	
	Reporting skill of project manager with contractor and subcontractor.	
CLRF	Influence of client's representative.	Saqib et al., 2008
	Client experience.	
	Owner's clear and precise definition of project scope & objectives.	
	Owner's risk attitude (willingness to take risks).	
	Client's ability to brief the project.	
	Client's ability to make appropriate decisions.	
	Client's ability to define roles clearly.	
K.C.	The project related internal documents or data files were easily accessible/available.	Songer and Molenaar, 1997
	The project related manuals or regulations were easily accessible/available.	
	The projected related professionals and experts were easily accessible for a meeting.	
	The professional databases or websites were easily accessible to acquire projected related knowledge.	
	Our colleagues and supervisors were easily accessible/available for sharing their valuable projected related knowledge.	
	We had an opportunity to hold an informal meeting with team members to share our projected related ideas/knowledge.	
	Team members were encouraged to keep the records of working knowledge in a written form.	
	Team members were encouraged to keep the records of working knowledge as a standardized procedure.	
	Team members were encouraged to keep the records of working knowledge in the information system.	
	Team members were encouraged to update the work-related profiles for further use.	
	Team members were encouraged to store the work-related rules or regulations in a written format or information system.	
	Team members were encouraged to transfer their knowledge or experience to others.	
	Team members were encouraged to discuss and share their opinions and documents with colleagues.	
	Team members were encouraged to quickly respond and provide our team with the necessary information, documents, or techniques whenever they encountered problems.	
CSRF	The project was successful in meeting its functional performance of customers' expectations.	Shenhar et al., 1997
	The project was successful in meeting technical specifications as required by the customer.	
	The project was successful in fulfilling customers' needs.	
	The project was successful in solving customers' problems.	
	The customer is using the delivered product effectively/successfully.	
	Customers are satisfied with project deliverables.	
		

Management-related factors are coded as MGTRF, design-related factors as DRF, customer-related factors as CRF, project manager-related factors as PMRF, client-related factors as CLRF, tacit knowledge creation as TKC, explicit knowledge creation as EKC, and customer satisfaction as CSRF. In data screening stage, outliers and missing values were identified to ensure that there are no missing values and data has been entered accurately.

### Reasons for Using the Smart PLS

In the current study Smart PLS has been used for several characteristics.

- As it indicates the maximum variation of latent variable and indicators.
- Another reason of using PLS path modeling is that; it had been widely used in the marketing literature and widely accepted (Lim, 2008; Voola and O'Cass, 2010). As Smart PLS very popular because of it free availability to researchers and academics, user friendly interface and highly developed reporting features.
- As it is less restricted and do not require the normally distributed data (Fornell and Cha, 1994).
- As well as compared to other path technique PLS requires the minimum demand on measurement scale, sample size and residual distribution.
- PLS approach is useful for analyzing the causal relationship, as well as PLS path modeling is useful for theory confirmation, as well as the for theory development (Sarkar et al., 2001; Urbach and Ahlemann, 2010; Umrani et al., 2020).
- Another benefit of PLS specifically the Smart PLS as they allow us to estimate the measurement model as well as the structural model at the same time (Ringle et al., 2005).

## Results and Discussion

### Demographic Study

The demographic study is conducted using the SPSS Statistical Package for Social Science software. The demographic research provides details about the education level of the respondents, the Designation of the respondents, and the highest results according to different categories. The demographic study results are summarized (see Table 5).

**Table 5:** Respondent profile.

**Demographic variables**	**Values**	**Frequency**	**Percentage**
**Project name**			
	Polio	131	53.3
	Dengue	27	11
	MCH week	9	3.7
	Measles	5	2.0
	EPI	5	2.0
	T.B Dot Program	50	20.3
	School Health Program	4	1.6
	MTOT-MCH	15	6.1
	Total	246	100
**Education**			
	Metric	41	16.7
	FA/Fsc	86	35.0
	BA/BSc	44	17.9
	MBBS/FCPS	52	21.1
	R. Nursing	22	8.9
	6.00	1	0.4
	Total	246	100.0
**Your position in the project**			
	Top	25	10.2
	Middle	56	22.8
	Operational	165	67.1
	Total	246	100.0
**Your experience in project related job**			
	1–2 Yrs	87	35.4
	2–5 Yrs	35	14.2
	>5 Yrs	122	49.6
	Total	246	100.0
**Your nature of work in the current/last project**			
	Technical/engineering	2	0.8
	IT/MIS	0	0
	Finance	0	0
	HRM	138	56.1
	Other (please specify)	106	43.1
	Total	246	100.0
**Total workforce of the current project**			
	<100	224	91.1
	101–300	18	7.3
	301–1,000	1	0.4
	1,001–5,000	3	1.2
	>5,000	0	0
	Total	246	100.0

### Statistical Technique: An Introduction to SEM

SEM is a multivariate strategy and second-generation tool for examining the relationship between many variables. The sample descriptive analysis, reliability analysis using the statistical package for social sciences (SPSS) by Arkkelin (2014), and additional partial least square technique (Smart PLS 3.2.7) by Götz et al. (2010) were employed in the statistical analysis to assess the suggested model or for hypothesis testing.

### Model Validation

The first step evaluated the measurement model, which checks the reliability and validity, the primary criteria for determining the measure's goodness. The second stage examined the structural model to determine the relationship between the latent variables. The model is also known as the outside model. As in the current study, the Cranach alpha ranged from 0 to 1 (see Table 6), and the composite reliability ranged from 0.6 to 0.9 (see Table 6), both of which met Hair et al. (2012) criterion or threshold of reliability requirements of at least 0.6 and 0.7.

**Table 6:** Internal consistency of the final instrument.

**Variables**	**Cronbach's Alpha**	**Composite reliability**
Client related factors	0.810	0.839
Contractor related factors	0.824	0.876
Design related factors	0.665	0.778
Knowledge creation	0.857	0.882
MGT related factors	0.900	0.915
Project manager related factors	0.818	0.875
Project success	0.838	0.879

According to Chin (1998), the indicator reliability is measured by item loading, which should be >0.7; however, according to Hair et al. (2012), the indicator loading must be 0.50 or greater.

**Figure 2:**
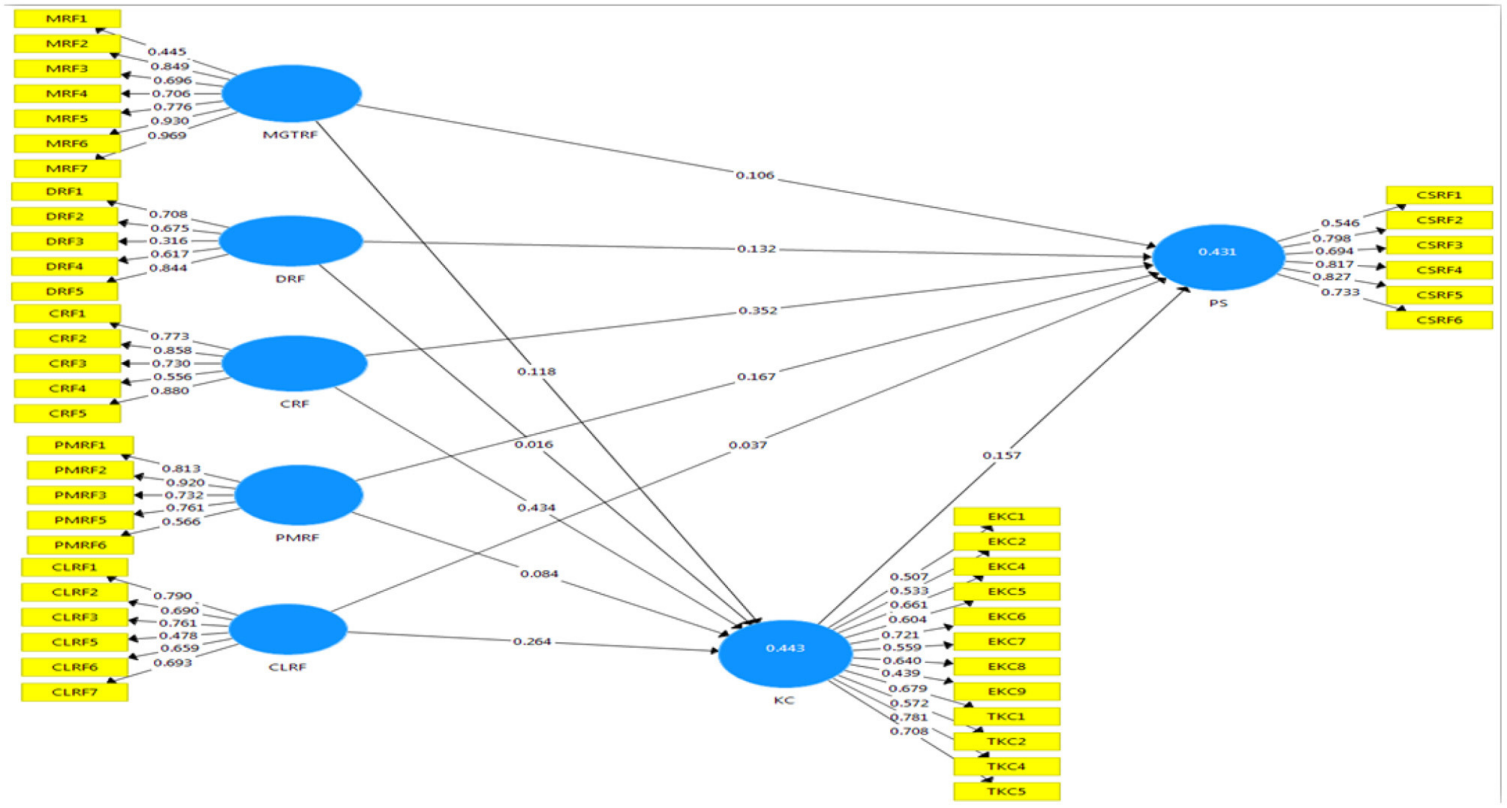
Measurement model indicating only indicators reliability. Source: Author's constructed.

The current study's results are displayed in the figure below (see Figure 2 and Table 7), and they show that item loading is satisfactory and meets the criteria, with values ranging from 0.437 to 0.969. According to Fornell and Larcker [61], convergent validity in Smart PLS 3.2.7 can be determined by the value of (AVE) average variance extracted, and the cut-off value for this measure is 0.50. The current study results for the (AVE) average variance extracted are provided in the table (see Table 8), with values ranging from 0.5 to 0.8.

**Table 7:** Factors loading.

**Variables**	**Factors loading**
**Management related factor**
MGRF1	0.445
MGRF2	0.849
MGRF3	0.696
MGRF4	0.706
MGRF5	0.776
MGRF6	0.930
MGRF7	0.969
**Design related factor**
DRF1	0.708
DRF2	0.675
DRF3	0.316
DRF4	0.617
DRF5	0.844
**Contractor related factor**
CRF1	0.773
CRF2	0.858
CRF3	0.730
CRF4	0.556
CRF5	0.880
**Project manager related factor**
PMRF1	0.813
PMRF2	0.920
PMRF3	0.732
PMRF5	0.761
PMRF6	0.566
**Client related factor**
CLRF1	0.790
CLRF2	0.690
CLRF3	0.761
CLRF5	0.478
CLRF6	0.659
CLRF7	0.693
**Explicit knowledge creation**
EKC1	0.507
EKC2	0.533
EKC4	0.661
EKC5	0.604
EKC6	0.721
EKC7	0.559
EKC8	0.640
EKC9	0.439
**Tacit knowledge creation**
TCK1	0.679
TCK2	0.572
TKC4	0.781
TKC5	0.708
**Project success**
CSRF1	0.546
CSRF2	0.798
CSRF3	0.694
CSRF4	0.817
CSRF5	0.827
CSRF6	0.733

**Table 8:** Convergent validity.

**Variables**	**AVE**
PS	0.55
CLRF	0.57
CRF	0.59
DRF	0.53
MGTRF	0.61
PMRF	0.58
KC	0.66

The last component that needs to be assessed is the discriminate validity, which the Fornell–Larcker criterion can be evaluated. Reason behind using this method is that, Fornell and Larcker criterion is the most widely used method for assessing the discriminate validity (Ab Hamid et al., 2017).

According to Fornell and Larcker (1981), the square root of AVE is used to test discriminative validity, and diagonal elements must be greater than the square root of AVE, as diagonal elements are the square root of AVE. The diagonal elements in the table below are greater than the diagonal elements (see Table 9).

**Table 9:** Fornell and Larcker criterion.

**Variables**	**CLRF**	**CRF**	**DRF**	**KC**	**MGTRF**	**PMRF**	**PS**
CLRF	0.754						
CRF	0.379	0.768					
DRF	0.151	0.297	0.728				
KC	0.491	0.584	0.198	0.812			
MGTRF	0.049	0.005	0.034	0.134	0.781		
PMRF	0.639	0.528	0.103	0.484	0.003	0.761	
PS	0.379	0.586	0.294	0.502	0.135	0.466	0.742

Following the evaluation of the measurement model, the structural model, also known as the inner model, is evaluated in the following stage. The first criterion in the SmartPLS 3.2.7 is to assesses each latent variable's coefficient of determination. The R square reflects how much the independent variable explains variance in the dependent variable.

Chin (1998) defines the R-square, stating that a value of 0.67 is considered significant, 0.20–0.33 is deemed to be average, and a value of 0.19 or lower is considered weak or indicates a poor relationship. The R-square values that met the Chin (1998) criteria are shown in the table (see Table 10).

**Table 10:** Coefficient of determination.

**Latent variables**	**R Square**	**Assessment**
Project success	0.431	Moderate
Knowledge creation	0.433	Moderate

### Normality Probability Plots of Variables

**Figure 3:**
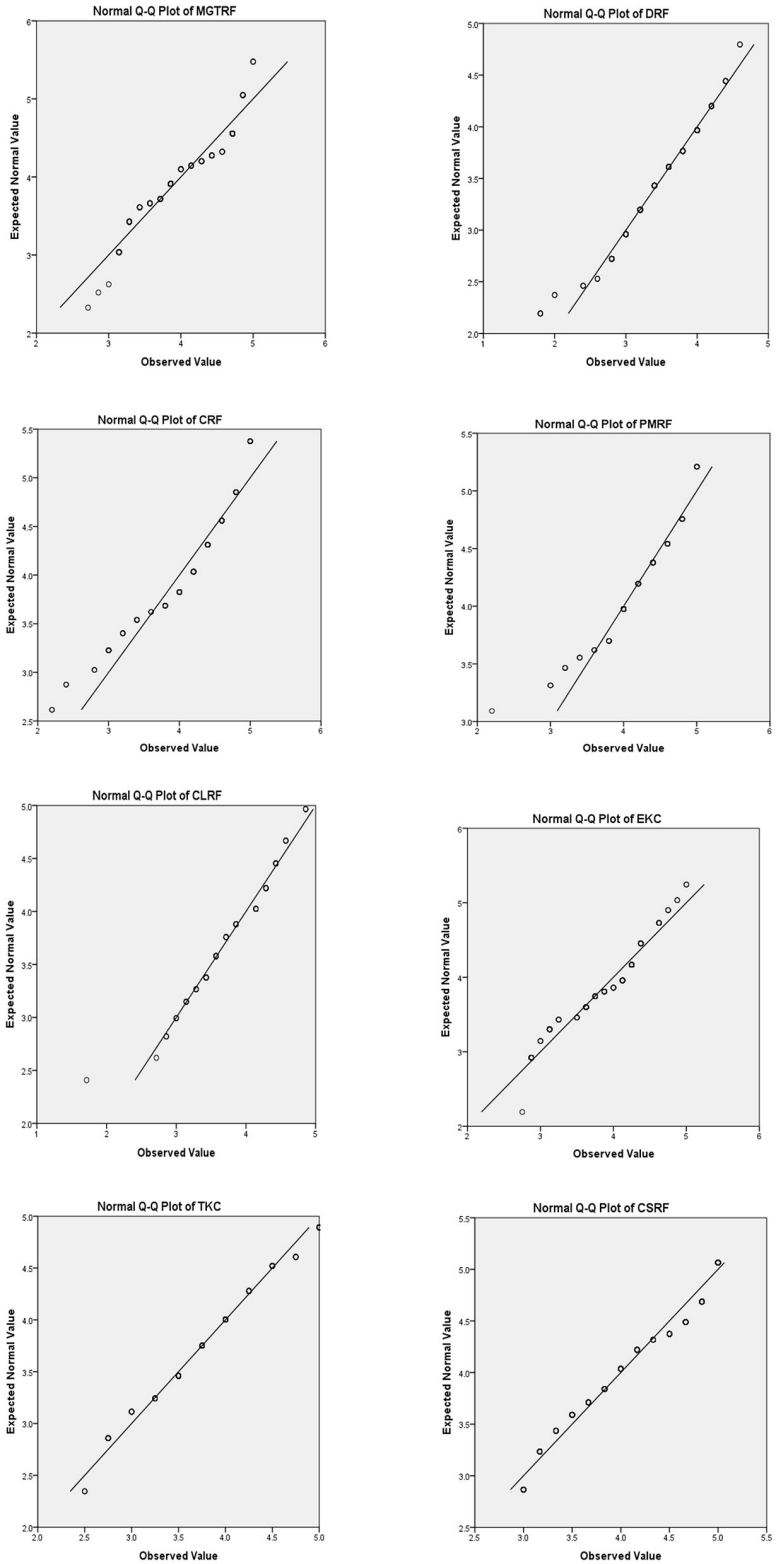
Normality probability plots of variables. Source: Author's constructed.

Furthermore, as illustrated in Figure 3, a normal probability plot (Q-Q plot) is utilized to assess the data's normality. As the instances go closer to the straight line, the normality plot for all variables shows an almost normal distribution.

### Multicollinearity

In SPSS, multicollinearity is calculated *via* Tolerance and VIF (Variance Inflation Factors). According to Arkkelin (2014), the cut-off value for tolerance is >0.10 and for VIF is <5. The values below indicate no multicollinearity issues (see Table 11).

**Table 11:** Multicollinearity.

**Constructs**	**Tolerance**	**VIF**
MGTRF	0.958	1.044
DRF	0.884	1.131
CRF	0.606	1.649
PMRF	0.516	1.939
CLRF	0.609	1.643
EKC	0.690	1.450
TKCRF	0.587	1.702

### Hypothesis Testing

T-values or significant levels are examined *via* bootstrapping. First, as shown in the table below, the direct relationship between the dependent and independent is examined (see Table 12). The findings show that the connections between CSFs, namely, management-related factors, design-related factors, contractor-related factors, project manager-related factors, client-related factors, and project success are positive, therefore supporting H1a, H1b, H1c, H1d, and H1e (see Table 12).

**Table 12:** Direct analysis (result after boostrapping).

**Path**	**Hyp**.	**(β)**	**Std. Error**	**T- value**	***P*-value**	**Results**
MGTRF->PS	H1a	0.251	0.119	2.109	0.027	Supported
DRF->PS	H1b	0.347	0.095	3.652	0.000	Supported
CRF->PS	H1c	0.322	0.139	2.312	0.024	Supported
PMRF->PS	H1d	0.305	0.125	2.440	0.018	Supported
CLRF->PS	H1e	0.339	0.110	3.082	0.006	Supported

### Mediating Analysis

Finally, the mediation effects were investigated using Smart PLS software. The current study's model predicts project success through critical success factors; nevertheless, the impacts manifested separately through different mediators, namely tacit and explicit knowledge creation.

According to Memon et al. (2018), when investigating models with several mediators, scholars must evaluate individual indirect effects rather than overall indirect effects. Nonetheless, current Smart PLS software updates feature a new option for examining multiple mediators known as ‘multiple specific indirect effects (mediation).' This function automatically calculates the indirect effect of each mediator, which can be mediation *via* implicit knowledge creation, explicit knowledge creation, or any variety of mediators.

As a result, evaluating models with many mediators is simplified by Memon et al. (2018). One of this study's contributions is the investigation of mediated interactions. The specific in-direct effect for the mediating variable is shown in Table 13.

**Table 13:** Mediating analysis (tacit and explicit knowledge creation) (result after boostrapping).

**Path**	**Hyp**.	**(β)**	**Std. Error**	**T- value**	***P*-value**	**Results**
MGTRF->TKC->PS	H2a	0.232	0.077	3.013	0.009	Supported
DRF-> TKC->PS	H2b	0.228	0.093	2.452	0.001	Supported
CRF-> TKC->PS	H2c	0.272	0.096	2.833	0.005	Supported
PMRF-> TKC->PS	H2d	0.237	0.096	2.469	0.017	Supported
CLRF->TKC->PS	H2e	0.455	0.123	3.691	0.000	Supported
MGTRF->EKC->PS	H3a	0.044	0.142	0.311	0.309	Not supported
DRF-> EKC->PS	H3b	0.124	0.133	0.935	0.248	Not supported
CRF-> EKC->PS	H3c	0.045	0.116	0.387	0.316	Not supported
PMRF->EKC->PS	H3d	0.061	0.115	0.530	0.279	Not supported
CLRF->EKC->PS	H3e	0.081	0.104	0.778	0.279	Not supported

The findings of the mediation test revealed that tacit knowledge creation mediated the association between critical success factors and project success, hence supporting H2a, H2b, H2c, H2d, and H2e. On the other hand, explicit knowledge creation does not mediate the relationship between critical success factors and project success; thus, H3a, H3b, H3c, H3d, and H3e were not supported.

## Discussion

Previous research (Dalkir, 2013; Todorović et al., 2015) found a gap that the current work covers. It demonstrates that knowledge creation acts as a bridge between critical success factors and project success. The present study further expands on the research paradigm that Todorović et al. (2015) developed by including knowledge creation as a mediator and project success as a dependent variable. Critical success factors exist in today's research arena concerning project success; however, the notion is still unclear on how these factors are associated with project success. The world has changed tremendously in the previous decade, and these changes are continuing at an accelerating rate. As a result of this project's ongoing challenges, we have failed to meet the needs and expectations of our customers. This study choose customer satisfaction as a project success dimension because their behavior directly impacts project success. The current studies concentrate on the critical success factors of health projects. Because the knowledgeable sector has previously gotten little attention. However, the collective data analysis results demonstrate a significant relationship between critical success factors and project success, found in previous literature (Pinto and Slevin, 1987; Odeh and Battaineh, 2002; Saqib et al., 2008).

In terms of tacit mediation, the findings showed a significant relationship between critical success factors and project success and that tacit knowledge creation had been positively influenced, supported by prior research. Tacit knowledge creation contributes to project success. As a result, tacit knowledge links success variables and project outcomes (Dalkir, 2013). According to Al-Hakim and Hassan (2014), mid-level managers impact knowledge creation execution. This tacit knowledge creation has been implemented successfully. It also boosts innovation and improves organizational/project performance. As a result, tacit knowledge creation mediate the relationship between mid-level management and project success.

According to the findings of this study, tacit knowledge creation mediates the relationship between critical success factors (management-related factors, design-related elements, contractor-related factors, project manager-related factors, and client-related factors) and project success. Hypothesis H2a, H2b, H2c, H2d, and H2e shows significant relationship. Because ours is a collectivist society. Our lives and jobs are belong to a collectivist society. Whenever an issue arises during project execution, we all interact, exchange our perspectives and thoughts, and work together to find the best solution. Throughout the process, tacit knowledge is articulating, no one seeks explicit knowledge. As a result, tacit knowledge creation mediates the relationship between CSFs and project success.

The findings revealed that explicit knowledge creation does not affect project success in explicit mediation. It does not mediates the relationship between critical success factors and project success. Previous studies in developed nations have shown that explicit knowledge creation considerably moderates the link between CSFs and project success. However, Pakistan is a developing country that prefers a collectivist approach. In a collectivist society, we typically utilize “We” instead of “I,” according to Hofstede's 5-dimension approach. Project team members embraced common responsibility rather than personal duty in the collectivist method. The organization's benefit is important for the people living in collective culture places.

They are unconcerned about their interests. When a project team member encounters an issue during project execution in a collectivist culture. A team member discusses their problem with the rest of the team. They called a formal meeting, and everyone shared their opinions and points of view based on their experiences. They can only conclude and solve the problem through this formal meeting.

As a result of this formal gathering, tacit knowledge is expressed, and no one seeks explicit information. Hypotheses H3a, H3b, H3c, H3d, and H3e are rejected since they do not demonstrate a significant association with project success.

## Conclusion

In project management, project success is an intriguing and essential topic. As in any competitive environment, project managers are motivated to maximize project success, and to that end, they employ various strategies to distinguish themselves from their competition. The current research examines impact of critical success criteria on project success. All of the variables in the research model were derived from past research. Data were acquired from the health programmes at DHQ hospital Attock and PIMS hospital Islamabad. After verifying the reliability and validity of research scales, the hypothesis was evaluated, revealing that all measurements were reliable.

The current study found that critical success factors (management-related factors, design-related factors, contractor-related factors, project manager-related factors, and client-related factors) strongly correlate with project success (see Table 9). It means that critical success factors are important predictors of project success. As a result, to get the most out of the project, managers and subordinates should concentrate on several critical success factors. It was also shown that tacit knowledge production mediates the relationship between critical success elements and project success, whereas explicit knowledge does not.

It is due to Pakistan's status as a developing country. It takes a collectivist approach rather than an individualist one. Individual interest is prioritized over community interest in a collectivist society. As a result, tacit knowledge rather than explicit knowledge is communicated. The current research can be described in two parts: marketing implications and theoretical contribution. The marketing application will investigate the practical applicability of recent study findings in today's project, whilst the theoretical contribution will fill a knowledge gap in the previous literature.

### Managerial Implication

- This study helps the project manager and subordinates discover essential elements for project success. To combat the severity of these factors, the manager should change their strategies. Managers should instruct their employees on generating tacit and explicit knowledge that may be used efficiently when a team member confronts a challenge.
- The research work assists project managers in developing strong team cohesion. The manager should encourage their project team members to speak with and share their ideas. Also, reply fast and supply their team with the essential information, documents, or procedures whenever they meet challenges.
- Project managers must also construct a structure within a project that generates both implicit and explicit knowledge. They should announce a reward scheme for their team members in incentives and allowances to accomplish this.
- The results suggest that one must gain continuous and sustained top management support before launching any knowledge management project. Leadership developed with transformational leadership traits is the core requirement to make the project successful.
- Top management of the organization is required to keep leadership development on top of the agenda for projects. In consultation with all stakeholders, a clear vision and objectives for knowledge management must be formulated and articulated. A well-developed knowledge management framework and knowledge cycle be adopted and communicated to all employees. A strategy about top-down and bottom-up learning and knowledge sharing may also be formulated and widely circulated before practically launching the knowledge management project.
- Project managers need to get people involved in Knowledge management processes that require continuous learning and development. Training also needs to be included in the knowledge management program. However, before launching any training program, identify knowledge management related competencies and behaviors through a knowledge audit. It will enable their people to build their knowledge processing capabilities and competencies and will realize the priority of the knowledge management project.

### Theoretical Contribution

- Previous studies only focused on the proposed concept of success analysis only. For example determination of CSFs and success criteria and then correlating CSFs and success criteria (Bhatti, 2005; Hyvari, 2006; Saqib et al., 2008). This study is going to examine the influence of critical success factors on project success through the mediation of knowledge creation.
- The current study also improves the research framework initially designed by [5], after adding the knowledge creation as a mediator and project success as a dependent variable.
- Little is known about the antecedents and consequences of critical success factors which can further lead to crucial project outcomes like time overrun and cost overrun, and there is still room for further exploration in this regard (Saqib et al., 2008; Todorović et al., 2015). So the current research adds to the database existing in project success literature.

### Recommendation to Practioner

- The health project manager should develop a system that focuses on the generation of implicit and explicit knowledge and the formulation of tacit and explicit knowledge.
- Coordination between senior management and project subordinates is lacking. Several staff members in the DHQ Attock hospital complained that upper management does not respond on time and coordinate adequately, causing project operations to be delayed. As a result, senior management must communicate with their subordinates on time and effectively.
- Vertical collectivism and centralization underpin the health initiative. We recommend that the health initiative follow horizontal collectivism. In which responsibility is distributed to each individual, each individual can decide on their behalf. Project activities will not be delayed as a result of these timely decisions.
- This research educates practitioners on the CSFs characteristics that influence project success in the health sector. And when these difficulties are adequately managed, many projects stand a reasonable chance of succeeding.
- This study is significant because top management will gain knowledge from their great coordination with their subordinates, which is the primary reason for project success. It makes the authorities comprehend how they can utilize various tactics to handle the essential success aspects in the project to complete, so that the work environment more comfortable and lead the project to success.
- It enables policymakers to understand better the impact of critical success factors on project success in the health sector, allowing new policies to be developed to address these critical success factors that may affect project performance.

### Limitation and Future Recommendation

The present study uses cross-sectional and quantitative research methods; thus, different methodologies are being used to predict behavior better. The information was gathered from two hospitals and can be expanded further. Because many other marketing elements may impact project success, but only a few were evaluated in this study, future studies should extend the other variables (project KPIs) to understand better, how and to what extent the project's success occurs. The current study only looks at mediation; thus, future research should look into other mediating variables (knowledge acquisition, knowledge application, knowledge transfer) and moderating variables (such as project experience) to better assess the results.

In the future, cross-sectional studies can address the issue of generalizability, with study samples drawn from various areas of Pakistan, particularly those previously out of reach due to financial restrictions and the risk of terrorist activity. Many other components, such as the industrial sector other than the health sector, might be expanded to broaden the research. Little is known about the causes and effects of critical success variables, leading to important project outcomes such as time and cost overruns. There is still an opportunity for future research in this area (Saqib et al., 2008; Todorović et al., 2015). As a result, the current study adds to the database of project success literature.

## Data Availability Statement

The original contributions presented in the study are included in the article/supplementary material, further inquiries can be directed to the corresponding author.

## Ethics Statement

Ethical review and approval was not required for the study on human participants in accordance with the local legislation and institutional requirements. Written informed consent from the patients/participants was not required to participate in this study in accordance with the national legislation and the institutional requirements.

## Author Contributions

The idea of the original draft belongs to SaN. SaN wrote the introduction, literature review, and empirical outcomes sections. MA, HH, and SiN helped collect and visualize data of observed variables. SaN, MVA, and KA constructed the methodology section in the study. All authors contributed to the article and approved the submitted revised version.

## Funding

This work was supported by a grant of the Romanian Ministry of Education and Research, CNCS - UEFISCDI, project number PN-III-P4-ID-PCE-2020-2174, within PNCDI III.

## Conflict of Interest

The authors declare that the research was conducted in the absence of any commercial or financial relationships that could be construed as a potential conflict of interest.

## Publisher's Note

All claims expressed in this article are solely those of the authors and do not necessarily represent those of their affiliated organizations, or those of the publisher, the editors and the reviewers. Any product that may be evaluated in this article, or claim that may be made by its manufacturer, is not guaranteed or endorsed by the publisher.
